# Optimisation of the production of a selenium-enriched polysaccharide from *Cordyceps cicadae S1* and its structure and antioxidant activity

**DOI:** 10.3389/fnut.2022.1032289

**Published:** 2022-10-20

**Authors:** Wanwan Zhuansun, Jun Xu, Hengzhao Liu, Ying Zhao, Lulu Chen, Shufang Shan, Shiqin Song, Haoyu Zhang, Tingting Dong, Huawei Zeng, Qinxiang Xu

**Affiliations:** ^1^Anhui Province Key Laboratory of Pollutant Sensitive Materials and Environmental Remediation, School of Life Sciences Huaibei Normal University, Huaibei, China; ^2^R&D Center of Anhui Kouzi Distillery Co., Ltd, Huaibei, China; ^3^Key Laboratory of Se-enriched Products Development and Quality Control, Ministry of Agriculture and Rural Affairs, Ankang, China; ^4^National-Local Joint Engineering Laboratory of Se-enriched Food Development, Ankang R&D Center for Se-enriched Products, Ankang, China

**Keywords:** *Cordyceps cicadae*, selenium-enriched, polysaccharide, response surface optimization, antioxidation activity

## Abstract

The fermentation medium of a newly identified *Cordyceps cicadae S1* was optimized by response surface methodology, with the optimal medium containing sucrose (80 g/L), yeast powder (60 g/L), KH_2_PO_4_ (5 g/L), MgSO_4_·7H_2_O (1 g/L) and Na_2_SeO_3_ (0. 1 g/L). Under these conditions, the extracellular polysaccharide yield was 8.09 g/L. A novel selenium-enriched polysaccharide (PACI-1) was isolated from *Cordyceps cicadae*, purified and identified as a homofructose polysaccharide with a low average molecular weight of 9.95 × 10^3^ Da. The fine structure of PACI-1 was analyzed using NMR, CD, and AFM. Additionally, the *in vitro* antioxidant results showed that the PACI-1 had stronger antioxidant capacity than natural polysaccharides. These results provided a candidate strain for producing selenium polysaccharide and a new polysaccharide from *C. cicadae*, which showed good antioxidant activity.

## Introduction

*Cordyceps cicadae*, which belongs to the Clavicipitaceous family and *Paecilomyces* genus, is an entomogenous fungus (1). This medicinal fungus produces numerous primary metabolites in its fermentation liquid, including polysaccharides, proteins, nucleotides, adenosine, and ergosterol (2–4). Polysaccharides, the most abundant component in *C. cicadae* fermentation broth, have good antioxidant activity (5). Polysaccharides have been investigated to fulfill the purpose of raising their uses for antioxidant activity with removing free radicals (6, 7), thereby reducing the risk of ischaemic and cardiovascular diseases, Alzheimer's disease, cancer, Parkinson's disease etc. (8).

Selenium is an essential trace element for humans and animals. It can only be obtained from food and cannot be synthesized independently by the body. The daily recommended intake for adults is 60 μg (9). Selenium is a cofactor of more than 30 enzymes and a key component of selenoproteins. Selenium affects the functions of several body systems, including the central nervous system, endocrine system, immune system, and cardiovascular system (10). In the body, organic selenium compounds enhance immunity, exert anti-aging effects, prevent cardiovascular and cerebrovascular diseases, and inhibit cancer cell metastasis (11, 12). Today, billions of people worldwide live in selenium-deficient areas, mainly in China, New Zealand, and Europe (13). Accordingly, there has been increasing research attention on selenium compounds. Because inorganic selenium compounds have the disadvantages of high toxicity and unstable biological activity (14), obtaining high-yield organic selenium compounds is a research hotspot.

Selenium polysaccharides are a good selenium supplement as they combine the biological activities of selenium and polysaccharides. It has been reported that fungi can use inorganic selenium sources in the culture medium to convert exopolysaccharides into selenium-enriched exopolysaccharides in the process of liquid fermentation (15). For example, Alvandi added Na_2_SeO_3_ to the liquid fermentation medium of *Fomes fomentarius* to produce extracellular selenium polysaccharide. The modified extracellular selenium polysaccharide showed significant antioxidant activity and good antibacterial activity (16). In the present study, the medicinal fungus *C. cicadae* was taken as the research subject. Sodium selenite was added to its fermentation medium and the biotransformation method was used to synthesize selenium polysaccharide with the aim of obtaining a high-yield organic selenium compound. The experiment was divided into two stages. First, the components of the selenium-containing *C. cicadae* medium were optimized by response surface methodology to obtain the maximum yield of selenium polysaccharide. Second, the polysaccharide in the fermentation medium was isolated and purified and its structure was determined by high-performance liquid chromatography (HPLC), atomic force microscopy (AFM), Fourier-transform infrared spectroscopy (FT-IR), circular dichroism (CD), and nuclear magnetic resonance spectroscopy (NMR). Furthermore, its biological activity was studied, and the relationship between its structure and biological activity was preliminarily analyzed.

## Materials and methods

### Materials

The *Cordyceps cicadae S1* strain was screened and collected in the laboratory. Potato dextrose agar (PDA) was purchased from Best Biotechnology Co., Ltd. (Hangzhou, China); 1,1-diphenyl-2-pyridyl hydrazide (DPPH) and 2,2'-diazo-bis-3-ethylbenzothiazoline-6-sulphonic acid (ABTS) were purchased from Zhiji Biotechnology Co., Ltd. (Shanghai, China). The dialysis membranes, DEAE-52 cellulose anion exchange column, and Sephadex G-100 were purchased from Shanghai Qite Analytical Instrument Co., Ltd. (Shanghai, China). Other chemical reagents and solvents were of analytical grade and provided by Sinopharm Chemical Reagent Co., Ltd. (Shanghai, China).

### Identification of strains and determination of optimal medium conditions

#### Identification of strains

The *S1* strain was isolated from *Cordyceps cicadae* in the laboratory and inoculated into PDA medium by the plate scribing method. *C. cicadae* was cultured in a 28°C incubator for 5 days and the colony morphology was observed. DNA extraction, PCR amplification, fragment purification, and sequencing were conducted as previously reported (17). The obtained sequences were compared by BLAST and the phylogenetic tree was constructed using MEGA-X. The phylogenetic relationship between the isolated strains and the near-source strains registered in the GenBank database was analyzed.

#### Microbial cultivation

After 5 days of culture, the strain was used as the seed and inoculated into the seed culture medium (glucose 50 g/L, maltose 50 g/L, yeast powder 3 g/L, K_2_HPO_4_ 3 g/L, MgSO_4_·7H_2_O 0.2 g/L and pH 5.5), then cultured at 28°C with shaking (180 rpm) for 3 days. The cultured seed was inoculated into the liquid fermentation medium (fructose 20 g/L, yeast powder 20 g/L, K_2_HPO_4_ 1 g/L, MgSO_4_·7H_2_O 3 g/L, sodium selenite 0.05 g/L) at 10% (v/v). The liquid fermentation was carried out at 28°C and 180 rpm for 3 days.

#### Extraction and characterization of polysaccharides

The polysaccharide in the fermentation broth was extracted according to the method published by Qiao et al. (18). The carbohydrate content was determined by the phenol–sulfuric acid method with glucose as the standard. (19).

#### One-factor-at-a-time experiments

The fructose (carbon source) in the initial fermentation medium was successively replaced with glucose, maltose, lactose, and sucrose. The yeast powder (nitrogen source) in the initial fermentation medium was successively replaced with potassium nitrate, ammonium sulfate, beef extract, and peptone. The initial concentrations of both the carbon and nitrogen sources were 20 g/L. The carbon source and nitrogen source were tested at 20, 40, 60, 80, and 100 g/L. The concentration of MgSO_4_·7H_2_O was tested at 0, 1, 2, and 3 g/L. The concentration of K_2_HPO_4_ was tested at 0, 1, 3, 5, 7, and 9 g/L. The concentration of sodium selenite was tested at 0, 0.025, 0.050, 0.075, 0.10, and 0.125 g/L. Subsequently, the seed liquid was inoculated into the designed fermentation medium and cultured for 3 d under each set of fermentation conditions. Determination of the best condition for each tested parameters using the content of extracellular polysaccharide as an indicator.

#### Plackett-Burman (P-B) design

In the single-factor test, the influences of the concentration of sucrose, yeast powder, dipotassium hydrogen phosphate, potassium ions and magnesium ions were determined. Positive (+1) and negative (−1) levels of these five influencing factors were trialed, with the positive level of each factor being twice the negative level. The Plackett-Burman test was created in Minitab17 and repeated three times to determine the significance of each factor on the culture conditions (20).

#### Box-Behnken design

Based on the results of the P-B experiment, taking sucrose (X_1_), yeast powder (X_2_), and K_2_HPO_4_ (X_3_) as the independent variables and the yield of *C. cicadae* as response value (Y), response surface optimisation modeling was performed. The design of each factor level is shown in Table 1.

**Table 1:** Experimental design of RSM with 3 factors and 3 levels.

**Level**	**Factors**		
	**X_1_(Sucrose)**	**X_2_(Yeast powder)**	**X_3_(K_2_HPO_4_)**
−1	60	60	3
0	80	80	5
1	100	100	7

### Extraction and purification of extracellular polysaccharide from *C. cicadae S1*

The method of Xu et al. (21) was used with modification. 1,000 mL of *C. cicadae* fermentation broth was concentrated to 200 mL at 55°C using a rotary evaporator (Yarong re-2000b, Shanghai, China), then precipitated with absolute ethanol (600 mL) at 4°C for 12 h and centrifuged at 4,000 rpm for 10 min to collect the precipitate. The precipitate was dissolved in 100 mL of ultrapure water and the protein was removed from the solution using Sevag reagent (1-butanol/trichloromethane, 1:4 v/v). The remaining polysaccharide solution was dialysed using a 3,500 Da dialysis bag for 48 h and then precipitated with three times the volume of absolute ethanol for 12 h. The precipitate was freeze-dried in a freeze dryer (BoYiKang FD-1A-50, Beijing, China) to obtain the crude polysaccharide (PACI).

PACI was redissolved in pure water to a final concentration of 0.1 g/mL and then purified using a DEAE-52 column (2.6 × 30 cm) which was equilibrated with pure water. Then, 3 mL of the PACI solution was loaded onto the column, which was eluted with pure water and a step gradient of 0.1 to 0.3 M sodium chloride at a flow rate of 0.5 mL/min. Eluent (6 mL) was automatically collected in each tube. Trace detection was performed using the phenol-sulfuric acid method. PACI was divided into two peaks and the main fractions of each peak were freeze-dried to obtain a solid powder. A solution of the main fractions was purified using a Sephadex G-100 column (2.6 × 50 cm) with pure water as the eluent at a flow rate of 0.5 mL/min. Then, the main fraction was filtered using 8,000 Da molecular mass membranes to desalt. The quantity of polysaccharide was determined by the phenol-sulfuric acid method. After freeze-drying, one fraction (PACI-1) was obtained.

### Structural analysis of PACI-1

#### Molecular weight (Mw) determination

The uniformity and molecular weight of PACI-1 were determined by HPLC (Agilent 1260 series, Agilent Technologies, USA) with an evaporative light-scattering detector (ELSD) and ultrahydrogel 250 column (7.8 × 300 nm, Waters Corp., USA). The injection volume was 20 μL of 2 mg/mL sample solution, the mobile phase was ultrapure water, the flow rate was 1 mL/min, and the column temperature was 35°C. The T-series dextran standard was used to construct the standard curve, which was used to determine the molecular weight of PACI-1 (3).

#### Monosaccharide composition analysis

The monosaccharide composition of PACI-1 was determined by HPLC with PMP pre-column derivatisation, as previously reported (22). PACI-1 (10 mg) was hydrolysed with 2 M trifluoroacetic acid solution at 120°C for 6 h, and the excess trifluoroacetic acid solution was removed by a rotary evaporator. At 70°C, the obtained hydrolysate was derived with 0.5 M PMP in methanol and 0.3 M aqueous NaOH solution for 30 min, then neutralized with 0.3 M HCl. The derivatives were separated by HPLC equipped with a ZORBAX eclipse XDB-C18 column (4.6 × 250 mm) and a DAD detector. The column temperature was 30°C, the mobile phase was acetonitrile and ammonium acetate, and the flow rate was 1.0 mL/min.

#### FT-IR spectroscopy

Dried PACI-1 (1 mg) was mixed with 100 mg of dried KBr and pressed into a disk. FT-IR spectra were recorded in the range of 4,000–500 cm^−1^ (Spectrum 100 FT-IR, Thermo Fisher Scientific, USA) (23).

#### Circular dichroism (CD)

The CD spectrum (J-815, JASCO, Japan) of 0.5 mg/mL polysaccharide was recorded with a 1 cm path length. The CD spectrum accumulated at a rate of 50 nm/min from 190 to 400 nm. The bandwidth was 2.5 nm in this range (24).

#### Molecular morphology observation by AFM

Atomic force microscopy (AFM) was employed to observe the molecular morphology of PACI-1. PACI-1 was dissolved in pure water (1 × 10^−3^ mg/mL), dried on freshly cleaved mica in a dryer for 2 h and then observed using AFM (Hitachi High-Tech company, China) (25).

#### NMR spectroscopy

The PACI-1 (20 mg) were re-dissolved in 0.5 mL D_2_O, and then trans- ferred into 5 mm NMR tube for testing. The analysis was performed using an NMR analyzer. (VANCE-600, Bruker Inc., Rheinstetten, Germany)(23).

### Antioxidant activity of PACI-1

#### DPPH scavenging activity

The DPPH radical scavenging ability of PACI-1 was determined using the method of Sharma et al. (26) with modification. 2 mL DPPH was added to PACI-1 solutions (2 mL) of different concentrations (2, 4, 6, 8, 10 mg/mL). After reaction in the dark for 30 min, the absorbance value (A_j_) was measured at 517 nm. The DPPH free radical solution was replaced with absolute ethanol to determine A_i_ and the PACI-1 solution was replaced with absolute ethanol to determine A_c_. Ascorbic acid was used as the positive control. Each test was performed in triplicate. The equation to calculate DPPH radical scavenging capacity is:


$$\begin{array}{l} \text{Scavenging rate }\left(\text{\%}\right)=\left[1-\left({\text{A}}_{\text{j}}\;\times \;{\text{A}}_{\text{i}}\right)/{\text{A}}_{\text{c}}\right]\;\times \;100 \end{array}$$

#### ABTS scavenging activity

The ABTS radical scavenging activity of PACI-1 was determined according to the method of Zeng (8). ABTS (4 mL) and potassium persulphate solution were added to PACI-1 sample solutions (400 μL) of different concentrations (1, 2, 4, 6, 8, 10 mg/mL). The mixture was shaken evenly and allowed to react at room temperature for 6 min. Then, the absorbance was measured at 734 nm to determine A_0_. Distilled water was used as the blank and ascorbic acid was used as the positive control to determine A_1_. Each test was performed in triplicate.

The equation to calculate ABTS radical scavenging capacity is:


$$\begin{array}{l} \mathrm{Scavenging}\text{rate}\left(\%\right)=\left({\text{A}}_{0}\times {\text{A}}_{1}\right)/{\text{A}}_{1}\times 100 \end{array}$$

### Statistical analysis

All results are expressed as the mean value of at least three replicates ± standard deviation (S.D.). The data obtained were subjected to One-way analysis of variance (ANOVA) using IBM SPSS Statistics 21. A *p*-value of < 0.05 was considered statistically significant.

## Results

### Morphological identification

**Figure 1:**
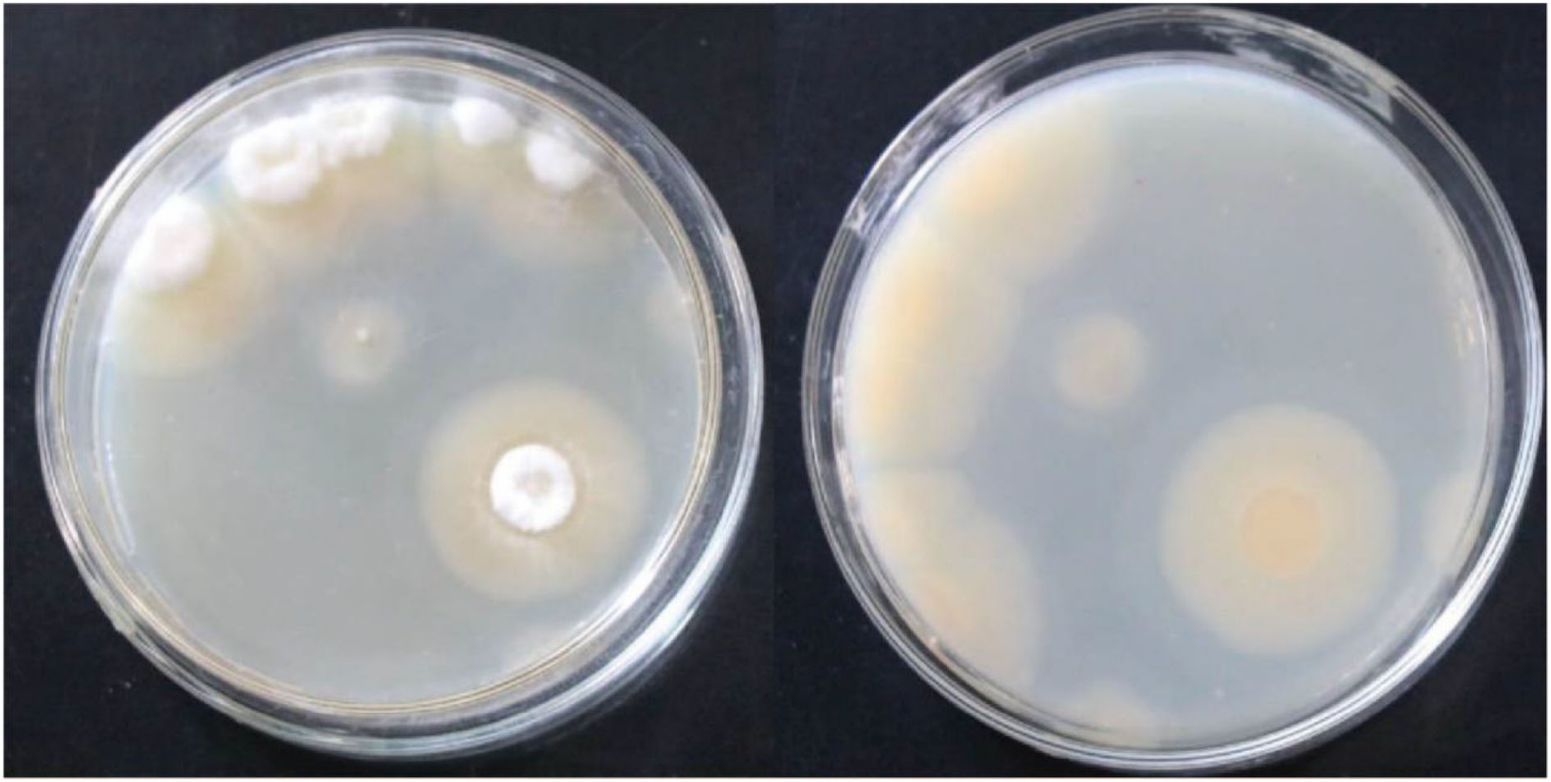
Frontal and reverse colony morphology of the strain *S1* after culturing on a PDA medium plate at 30°C for 144 h.

On PDA medium, the colonies were round with neat edges. They were flocculent in the early stages and powdery in the later stages. The colonies were light yellow on the front and the back. Aerial hyphae and vegetative hyphae were present (Figure 1). Based on morphology, the strain was preliminarily identified as mold.

### Molecular systematics and phylogenetic analysis

**Figure 2:**
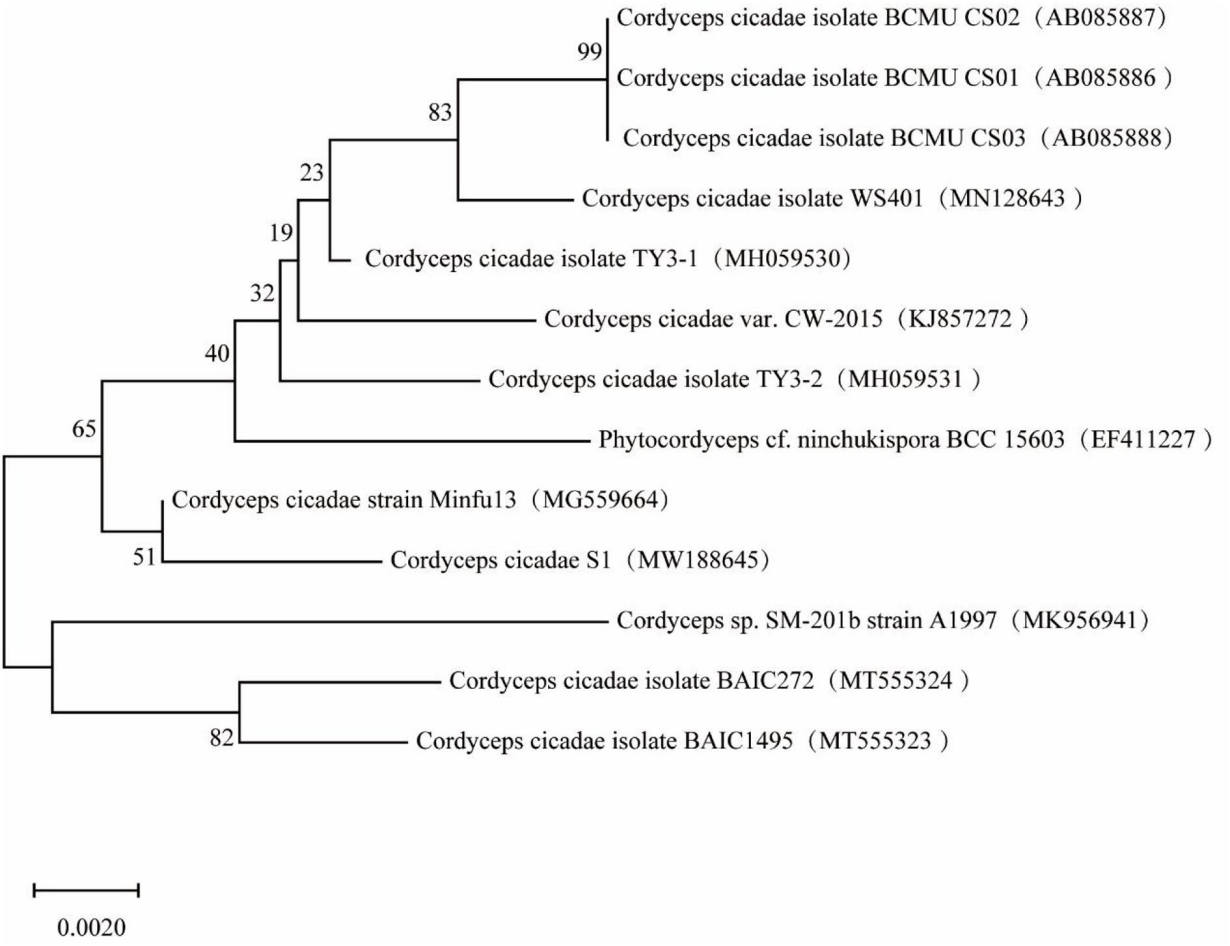
Phylogenetic tree based on strain *S1* sequence.

A phylogenetic tree of the *S1* sequence was constructed (Figure 2). The sequence for strain *S1* (Genbank Accession no. MW188645) had the highest similarity with *C. cicadae* strain *minfu13* (99.82%). Therefore, the strain should be classified as *C. cicadae*. In combination with the morphology and molecular systematics results, it was determined that the strain was *C. cicadae* and it was named *S1*.

### Single-factor test results

**Figure 3:**
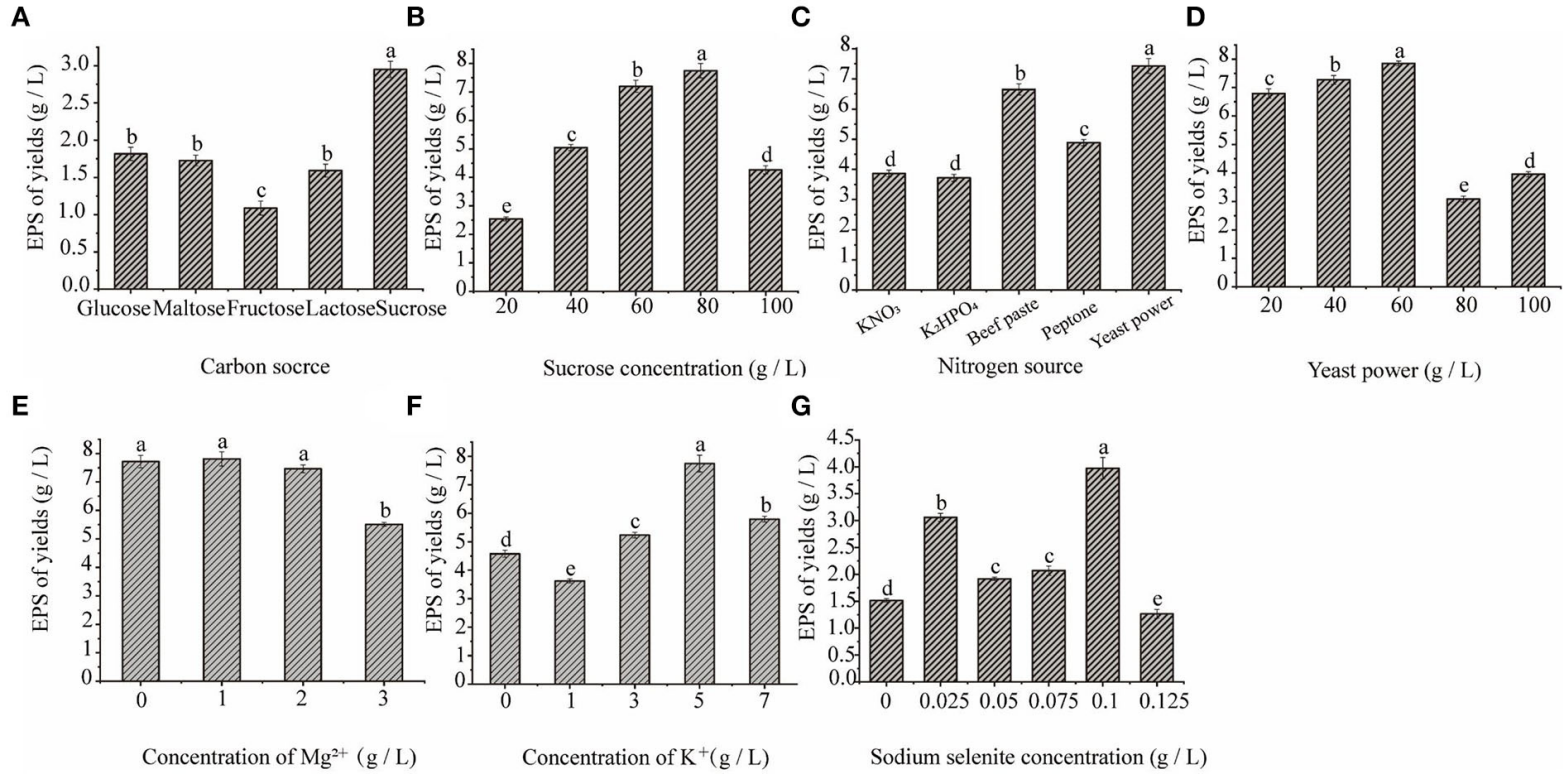
The effect of carbon source **(A)**, sucrose concentration **(B)**, nitrogen source **(C)**, yeast powder concentration **(D)**, magnesium ion concentration **(E)**, potassium ion concentration **(F)**, and sodium selenite concentration **(G)** on *C. cicadae* polysaccharide yield.

Figures 3A–G shows that when the concentration of sodium selenite was 0.1 g/L, the maximum yield of extracellular polysaccharides in *C. cicadae* was 3.97 g/L. As the concentration of sodium selenite increased, the polysaccharide yield decreased; therefore, the optimum concentration of sodium selenite was 100 mg/L. Among the five different carbon sources selected, when the carbon source was sucrose, the yield of polysaccharide was the highest (2.95 g/L); this was significantly higher than with the other four carbon sources. Sucrose is composed of the monosaccharides glucose and fructose, both of which are hexoses and can participate directly in the glycolysis and pentose phosphate pathways in the fermentation process of *C. cicadae*. This improved the carbon conversion efficiency of the reaction process and thereby improved the yield of extracellular polysaccharides (27). However, a further increase in the sucrose concentration inhibited the growth of bacteria. Too much sugar increased bacterial respiration and decreased dissolved oxygen in the fermentation broth, resulting in insufficient oxygen to meet the metabolic demands of the bacteria and affecting the synthesis of metabolites, including polysaccharides (28). Therefore, the optimum carbon source concentration was 8%.

Among the five different nitrogen sources, the highest yield of polysaccharide was 7.42 g/L after the addition of yeast powder, which was significantly higher than with the other nitrogen sources. Yeast powder is an organic nitrogen source, which is more conducive to improving the yield of medicinal fungal polysaccharides than inorganic nitrogen sources. Organic nitrogen sources are rich in proteins, polypeptides, free amino acids, and a small amount of fat, trace elements, and auxin, which are conducive to the growth of bacteria and the synthesis of various metabolites (29). When the concentration of yeast powder was 60 g/L, the maximum yield of polysaccharide was 7.85 g/L. The reason of yeast powder as the best nitrogen source may be that it can provide the necessary factors for microbial growth and can affect those enzyme activities involving the biosynthesis pathway of polysaccharide. However, as the concentration of yeast powder increased, the yield of polysaccharide decreased. This may have been because the higher concentrations of the nitrogen source led to the accumulation of nitrogen in the later stages of fermentation, which inhibited the synthesis of polysaccharides. Therefore, the optimum concentration of the nitrogen source was 60 g/L.

The addition of an appropriate amount of Mg^2+^ and K^+^ to the culture medium significantly promoted the yield of extracellular polysaccharide. Figures 3F,G show that when the concentration of Mg^2+^ was 1 g/L, the concentration of extracellular polysaccharide was the highest (7.81 g/L). As the Mg^2+^ concentration increased, the yield of extracellular polysaccharide decreased. Therefore, the optimum Mg^2+^ concentration was 1 g/L. K^+^ significantly promoted the production of extracellular polysaccharide by *C. cicadae* and the polysaccharide yield was the highest (7.75 g/L) when the concentration of K_2_HPO_4_ was 5 g/L. Metal ions can function as enzyme activators during the growth and polysaccharide production of *C. cicadae*, which is beneficial to bacterial growth and metabolism. However, high concentrations of Mg^2+^ and K^+^ are toxic to *C. cicadae* cells and negatively affect their growth and development.

In summary, the single-factor results for the *C. cicadae* liquid fermentation medium determined that the optimal conditions are as follows: 0.1 g/L sodium selenite, 80 g/L sucrose, 60 g/L yeast powder, 1 g/L MgSO_4_·7H_2_O, and 5 g/L K_2_ HPO_4_.

### Plackett Burman test results

The Plackett Burman test was used to determine the three factors that had the greatest impact on the polysaccharide yield from the liquid fermentation of *C. cicadae*. The test results are shown in Supplementary Table S1. Yeast powder and sucrose had a significant effect on the polysaccharide yield (*P* < 0.01), as did dipotassium hydrogen phosphate (*P* < 0.05). The order of significance was X_2_ (yeast powder) > X_1_ (sucrose) > X_3_ (K_2_HPO_4_). Therefore, the three factors with significant influence—yeast powder (X_2_), sucrose (X_1_) and K_2_HPO_4_ (X_3_)—were selected as the model factors for the subsequent response surface analysis.

### Response surface analysis

The response surface analysis results of the interaction among sucrose, yeast powder, and potassium hydrogen phosphate are shown in Supplementary Table S2. Minitab17 was used to analyse the results and a linear equation between the polysaccharide content (Y) and sucrose (X_1_), yeast powder (X_2_) and K_2_HPO_4_ (X_3_) was obtained: Y = 8.354-−0.019 X_1_ + 0.017 X_2_ + 0.047 X_3_−2.387 X${}_{1}^{*}$ X_1_−1.931 X${}_{2}^{*}$ X_2_−1.654 X${}_{3}^{*}$ X_3_−0.168 X${}_{1}^{*}$ X_2_−0.174 X${}_{1}^{*}$ X_3_ + 0.303 X${}_{2}^{*}$ X_3_(R^2^ = 0.9724).

A variance analysis was conducted to determine the reliability of this equation, with the results shown in Supplementary Table S3. The model value of *F* = 19.60 (*P* < 0.01) indicated that the model had a significant impact on the test results. The mismatch term of *F* = 0.28 (P > 0.05) had no significant impact on the test results. In the primary term, factor X_2_ had the greatest influence on the test results and the F values were in the order X_2_ > X_1_ > X_3_. X_12_, X_22_ and X_32_ in the secondary term had a significant impact, while no factors in the interactive term had a significant impact, indicating that there was no interaction between the factors. The adjusted model *R*^2^ value of 0.9228 indicated that 92.28% of the experimental results could be explained by the model.

**Figure 4:**
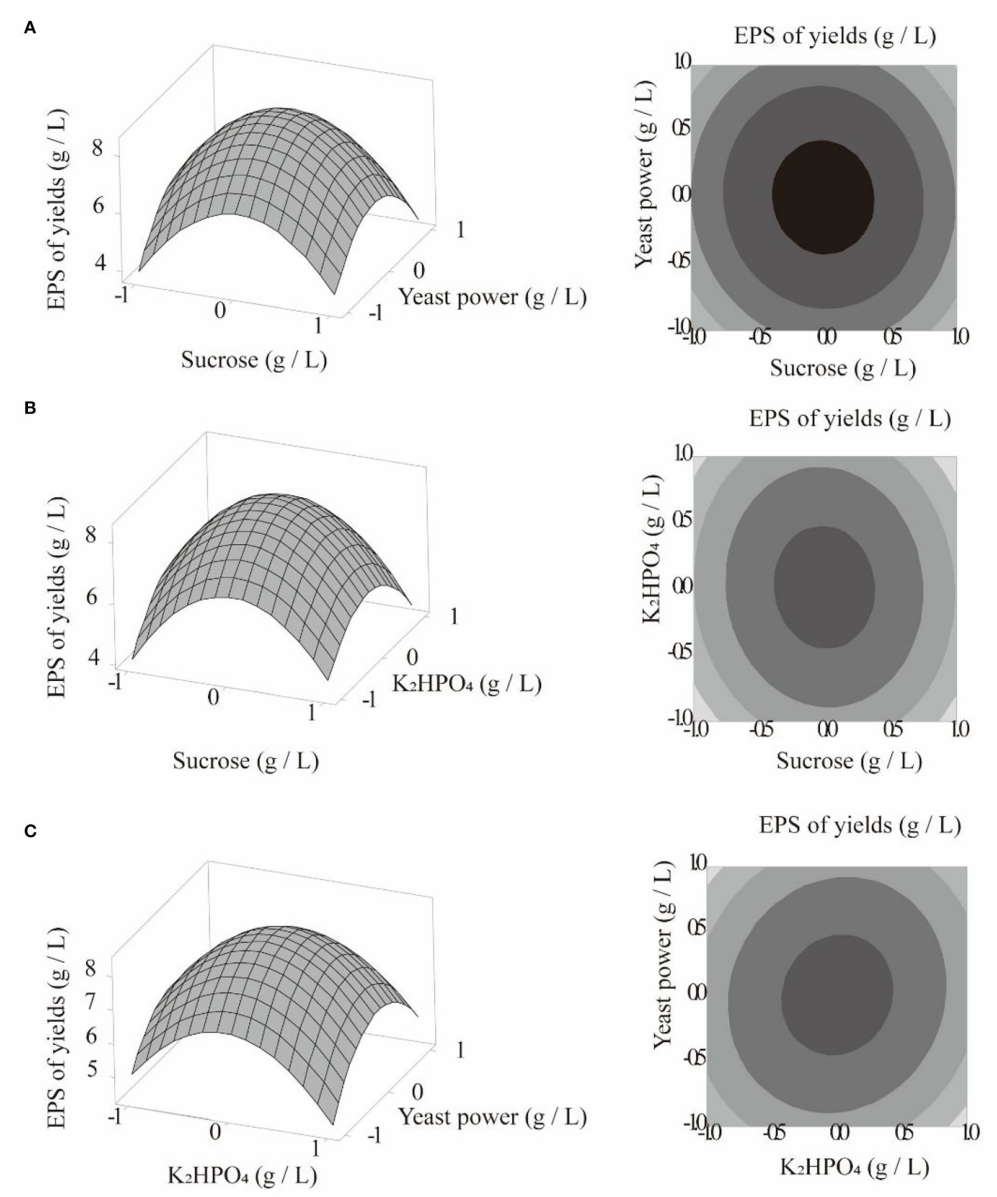
Curved surface plot and curved graph: polysaccharide yield, sucrose, and yeast powder **(A)**; polysaccharide yield, dipotassium hydrogen phosphate, and sucrose **(B)**; polysaccharide yield, dipotassium hydrogen phosphate, and yeast powder **(C)**.

This model was applied to predict the optimal medium composition. Taking one of the three factors as the central value and observing the three-dimensional response diagram of the remaining two factors, the influence of each factor on the response value was analyzed. The results are shown in Figures 4A–C. According to the Minitab17 results, when Y was maximal (8.3540 g/L), X_1_ = 0, X_2_ = 0.0101, X_3_ = 0.0101, sucrose = 79.9936 g/L, yeast powder = 60.0058 g/L, and K_2_HPO_4_ = 5.0153 g/L. For practical considerations, these values were revised to 80 g/L sucrose, 60 g/L yeast powder, and 5 g/L K_2_HPO_4_ for subsequent experiments. The average experimental yield of *C. cicadae* polysaccharide was 8.0942 g/L, which differed by only 3.1% from the predicted value. Therefore, this model has practical reference value.

### Purification and separation of PACI

**Figure 5:**
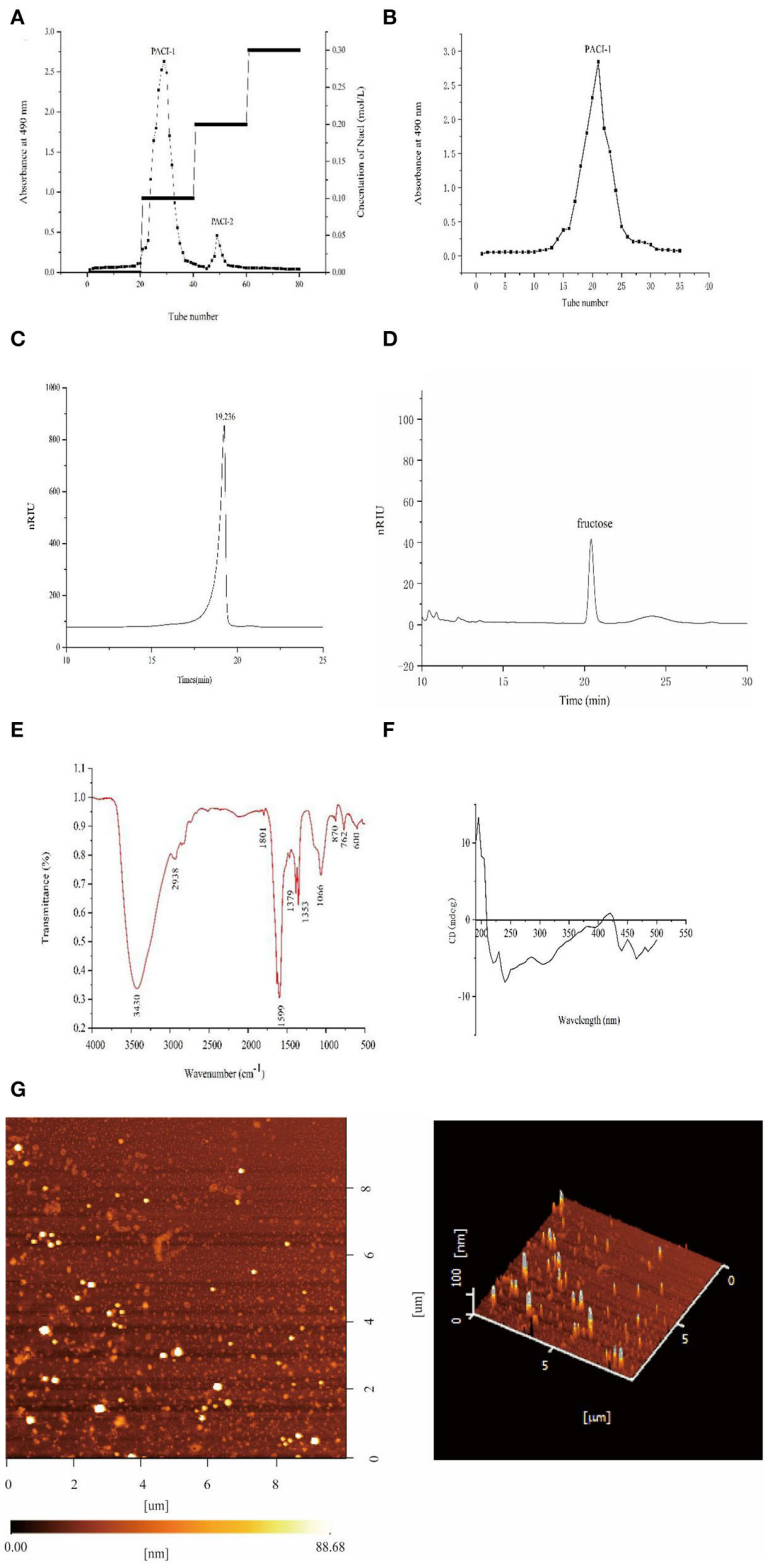
Structural analysis of PACI-1. DEAE-52 anion exchange elution curve **(A)**. G-100 gel elution curve **(B)**. High-performance liquid chromatography spectrum **(C)**. Monosaccharide composition **(D)**. IR spectrum **(E)**. CD spectrum **(F)**. AFM images of PACI-1 **(G)**.

After extraction of the crude polysaccharides from the fermentation broth by alcohol precipitation, PACI was separated using a DEAE-52 cellulose column and Sephadex G-100 gel column. According to the Figure 5A, there were three main fractions existed in it at the beginning of the purification process. The two fractions were then concentrated and named PACI-1 and PACI-2, respectively. After drying, the amount of PACI-2 was very small so was not studied further.

### Molecular weight and monosaccharide composition

The average molecular weight of PACI-1 was determined by HPLC. The HPLC spectrum contained a single symmetrical peak (Figure 5C), which indicated that the polysaccharide PACI-1 obtained after separation and purification had high purity and homogeneity. The calibration curve obtained from the dextran standard was log Mw = −0.2422 X + 8.7833 (*R*^2^ = 0.9974). According to calculations based on the standard curve, the molecular weight of PACI-1 was 9.95 × 10^3^ Da. PACI-1 was determined to be a homopolysaccharide composed of fructose (Figure 5D).

### Infrared spectroscopy

The FT-IR spectrum identified the following structural characteristics of the polysaccharide (Figure 5E). The peak from 3,500 to 3,100cm^−1^ corresponded to the stretching vibrations of O-H and C-H, which are characteristic absorption peaks of sugars. The peak at 3,430 cm^−1^ indicated that the polysaccharide had obvious intermolecular hydrogen bonding (30). The peaks between 3,000 and 2,800 cm^−1^ corresponded to the stretching vibrations of carbohydrate C-H moieties and the absorption peak at 2,938 cm^−1^ corresponded to stretching vibrations of C-H (31). The absorption peak at 1,379 cm^−1^ corresponded to the deformation of =CH2 and the peak at 1,353 cm^−1^ corresponded to C-H bending vibrations. The absorption peak at 1,066 cm^−1^ may have been due to the overlap of ring vibrations with the tensile vibrations of the C-OH side group and vibrations of the C-O-C glycosidic bond, indicating the presence of pyranose (32). Additionally, the stretching vibration of the pyran skeleton was also detected at 600 cm^−1^ and the peak at 870 cm^−1^ indicated the presence of β-glycosidic bonds (33).

### Circular dichroism

The CD spectrum of PACI-1 comprised a large positive peak at 195 nm with a positive cotton effect, which was due to the presence of C-O and O-H groups in the polysaccharide molecule (Figure 5F). There was a negative cotton effect at 240 nm. The presence of both positive and negative cotton peaks between 190 and 250 nm was indicative of molecular asymmetry; that is, PACI-1 could readily form curls, folds, and three-strand spiral structures in aqueous solution (34).

### Atomic force microscopy

AFM images were obtained to determine the surface morphology and roughness of PACI-1. In AFM, as the height is not affected by the “widening effect,” only the length and width change at different measurement positions. Therefore, the height can be used as a reference to determine the true diameters of molecules. The height of PACI-1 was about 88.68 nm, much higher than 15–50 nm, indicating that PACI-1 had a three-strand helical structure (35). This was consistent with the CD results. Additionally, the height of PACI-1 was much higher than that of monosaccharide chains without selenium modification (0.1–1.0 nm) (36).

As seen in Figure 5E, PACI-1 had an irregular polymer particle morphology. No irregular linear molecular chain conformations were observed. This phenomenon indicated that there was cross-linking of the PACI-1 polysaccharide chains to form polymer particles. This may have been due to van der Waals forces between the polysaccharide chains or hydrogen bond interactions between the molecules, causing the polysaccharide chains to intertwine and form a spherical structure (34).

### NMR spectroscopy

**Figure 6:**
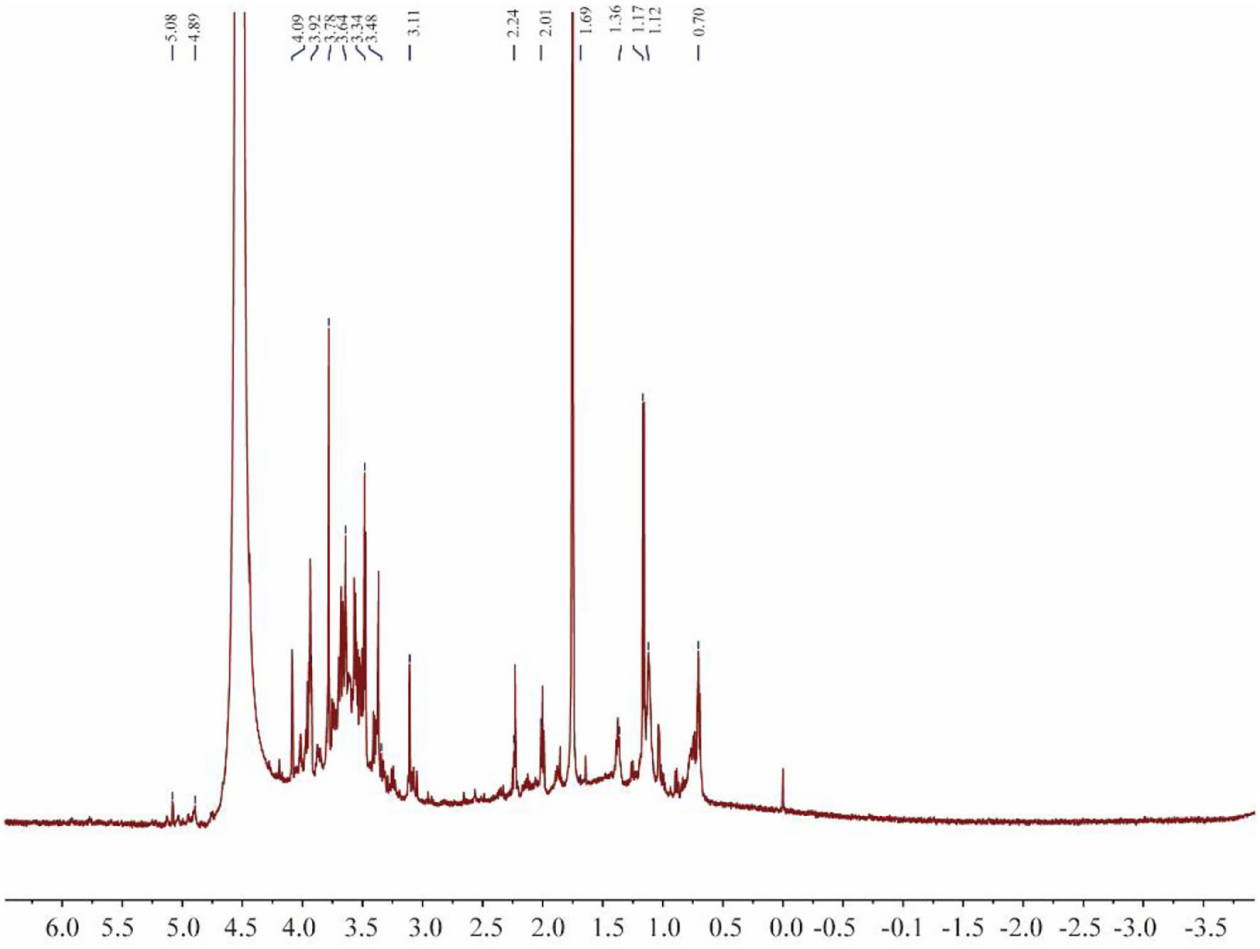
^1^H-NMR spectrum of PACI-1.

^1^H NMR can be used to determine the configuration of glycosidic bonds in polysaccharides (Figure 6). Generally, the α proton signal of α-type glycosidic bonds is at 5–6 ppm and the β proton signal of β-type glycosidic bonds is at 3–5 ppm (37). The ^1^H NMR spectrum contained a peak at 4.89 ppm, indicating that PACI-1 may contain β-type glycosidic bonds. Additionally, the main heterocephalic carbon proton signal of CPA-1 was at 3–4 ppm, indicating that PACI-1 mainly contained β-configured pyranoside bonds. This conclusion was the same as that obtained using infrared spectroscopy.

### *In vitro* antioxidant activity of PACI-1

**Figure 7:**
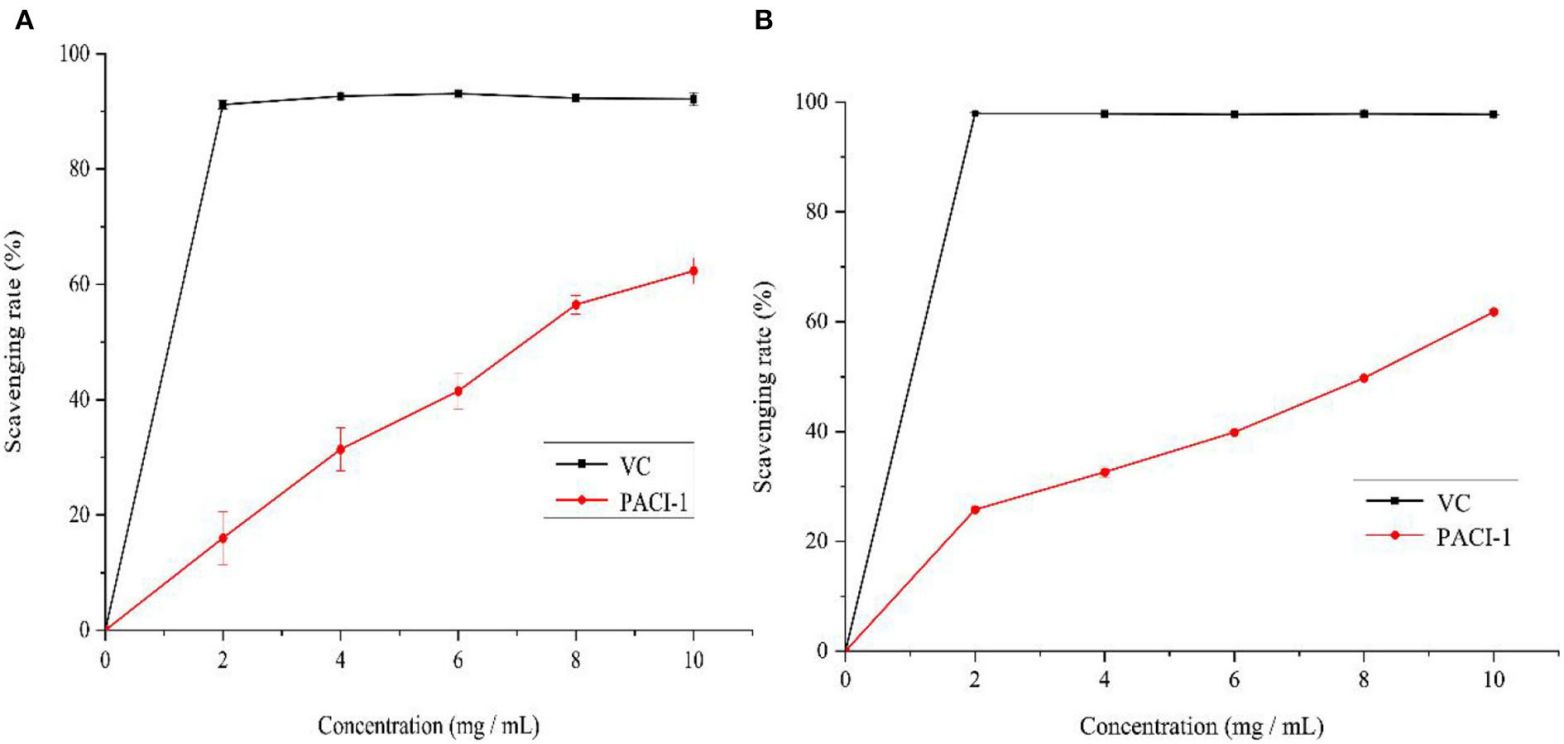
Scavenging effect of PACI-1 on DPPH **(A)** and ABTS radicals **(B)**.

The scavenging ability of PACI-1 for DPPH and ABTS free radicals was studied with Vitamin C as the positive control. The DPPH radical scavenging ability of PACI-1 is shown in Figure 7A. The free radical scavenging ability of PACI-1 increased as the polysaccharide concentration increased. At 8 mg/mL, the DPPH scavenging rate of PACI-1 reached 56.46%, which was higher than that of FVR-1 (47.38%), a natural polysaccharide component from *Flammulina velutipes* (38). As shown in Figure 7B, the scavenging ability of PACI-1 for ABTS free radicals had an obvious concentration dependence as the polysaccharide concentration increased. The IC50 value for the scavenging of ABTS free radicals by PACI-1 was 8.45 mg/mL, which was greater than the ABTS radical scavenging ability of the natural polysaccharide PKP-E-1-1 isolated from *Pinus koraiensis* (IC50 = 1.52 × 10^3^ mg/mL) (33). These results confirmed the antioxidant capacity of PACI-1, which increased as the concentration of PACI-1 increased.

## Discussion

After optimizing the medium formula, the amount of polysaccharide of 8.09 g/L produced by *C. cicadae* liquid fermentation was 642.20% higher than that without optimisation (1.09 g/L). This increase in polysaccharide yield was much higher than that obtained by Yang and Xu (21.70 and 50%) (39). Additionally, the optimized result was 1.41 times that obtained by Wang using shaking flask fermentation (5.71g/L) (40), and 16 times that obtained by Wang(486.16 ± 19.60 mg/L) (41). In the present study, after optimizing the components of the liquid medium, not only was the improvement in polysaccharide yield greater than those reported in other publications, but the yield was also relatively high. Therefore, this preliminary work provided an excellent approach to improving the polysaccharide yield.

The FT-IR absorption peaks near 605 cm^−1^ (Se=O), 760 cm^−1^ (C–O–Se), and 1610 cm^−1^ (O–Se–O) indicated that the selenite group underwent substitution on the polysaccharide (40). The FT-IR spectrum of polysaccharide component PACI-1 contained an absorption peak at 762 cm^−1^, which corresponded to Se-O-C stretching vibrations (42, 43). These changes indicated that PACI-1 had been selenised.

The chromatographic analysis revealed PACI-1 to be a homopolysaccharide composed of fructose with an average molecular weight of 9.945 × 10^3^ Da. As a comparison, the molecular weights of polysaccharides JCH-1 and JCH-2, purified from *Isaria cicadae Miquel*, were 3.09 × 10^4^ and 5.55 × 10^5^ Da (21), respectively, while the molecular weight of heteropolysaccharide (IPS1) from *Paecilomyces cicadae* was 2.40 × 10^6^ Da (44). Chen determined that the molecular weight of the extracellular polysaccharide W-CBP50 II in fermentation broth was 9.97 × 10^4^ Da (45). PACI-1, with its lower molecular weight of <1 × 10^4^ Da, would be classified as a low-molecular weight polysaccharide (33). The differences in the molecular weights of these polysaccharides may be related to the different sources, extraction methods, and purification steps for polysaccharides obtained from the fermentation liquids of different strains. Alternatively, the lower molecular weight of PACI-1 may have been caused by the addition of inorganic selenium (6, 46).

PACI-1 was determined to be a homopolysaccharide composed of fructose, which was inconsistent with previous experimental results (47). It was previously reported that the polysaccharide of *C. cicadae* mycelium was a heteropolysaccharide, with glucose, mannose, and galactose as the main components. Table 2 shows that the polysaccharides extracted from *C. cicadae* have been mainly heteropolysaccharides, while there are few reports in which the extracted polysaccharide was a homopolysaccharide (51). Therefore, this separation and purification study provides the first report on selenium-enriched syn-fructose polysaccharide.

**Table 2:** Monosaccharide composition of polysaccharides extracted from different samples.

**Sample**	**Polysaccharide fraction**	**Average molecular weight**	**Monosaccharide composition**	**References**
*P.cicadae TJJ1213*	IPS1	2.40 × 10^6^ Da	Mannose, glucose and galactose residues	(48)
	IPS2	6.79 × 10^5^ Da	Mannose, glucose and galactose residues	
*Isaria cicadae Miquel*	JCH-1	3.09 × 10^4^ Da	Glucose, mannose and galactose	(21)
	JCH-2	5.553 × 10^5^ Da	Glucose, mannose and galactose	
*P.cicadae ZJ001*	FPCPS		mannose, rhamnose, xylose and arabinose	(2)
*Thlaspi arvense L*	Se-PPS1	4.2 × 10^4^Da	Glucose, galactose, xylose and arabinose	(49)
	Se-PPS3	4.5 × 10^4^ Da	Glucuronic acid, rhamnose, galacturonic acid, glucose, galactose, xylose, and arabinose	
*C. gunnii*	SeCPS-ll	3.72 × 10^6^ Da	L-rhamnose,D-mannose, D-glucose and D-galactose	(50)
*Cordyceps militaris*	SeCPS-I	1.9 × 10^6^ Da	D-Mannose, D- Glucose, and D-Galactose	(43)
	SeCPS-ll	6.5 × 10^4^ Da	D-Mannose, D- Glucose, and D-Galactose	
	SeCPS-llI	1.6 × 10^4^ Da	D-Mannose, D- Glucose, and D-Galactose	

The antioxidant experiment conducted in this study showed that the selenium-enriched polysaccharide PACI-1 had a certain antioxidant capacity, which was higher than that of natural polysaccharides but lower than that of other selenium-enriched polysaccharides (52). It has been reported that low-molecular weight polysaccharides have high antioxidant activity. Therefore, the lower antioxidant activity of natural polysaccharide components FVRP-1 and PKP-E-1-1 compared to PACI-1 may have been due to their higher average molecular weights of 29,930 and 1.09 × 10^4^ Da, respectively. The antioxidant activity of polysaccharides is not related to just one factor but several (45), including the degree of branching, connections, conformation, and other factors (31). Therefore, the complex relationship between the polysaccharide structure and antioxidant activity needs further exploration and research to elucidate the reasons underpinning the activity of PACI-1.

## Conclusion

In this study, the components of the liquid fermentation medium of strain *S1* were optimized. The response surface results identified the optimal medium ratio at which the greatest yield of extracellular polysaccharide was obtained (8.09 g/L). A selenium source was added to the fermentation medium of strain *S1* to prepare the selenium polysaccharide and the extracellular polysaccharide in the fermentation broth was studied. The structure of component PACI-1 was analyzed by HPLC, FT-IR, CD, AFM, and NMR. The results showed that PACI-1 had an average molecular weight of 9.945 × 10^3^ Da in selenium- and fructose-enriched medium, and a three-strand helical-spherical structure in aqueous solution. Additionally, the antioxidant activity of PACI-1 was between those of the natural polysaccharides and selenium polysaccharides. However, the complete structure-activity relationship of PACI-1 remains unclear. Future studies will further explore the relationship between the bioactivity and structure of extracellular polysaccharides from *C. cicadae*.

## Data availability statement

The original contributions presented in the study are included in the article/Supplementary material, further inquiries can be directed to the corresponding author.

## Author contributions

WZ: conceptualization, methodology, software, investigation, formal analysis, and writing—original draft. JX: data curation and writing—original draft. HL: visualization and investigation. YZ: resources. LC: Supervision. SSh: software. SSo: validation. HZh: visualization. TD: data analysis. HZe and QX: conceptualization, funding acquisition, resources, and supervision. All authors contributed to the article and approved the submitted version.

## Funding

This work was financially supported by the Open Project Program from Key Laboratory of Se-enriched Products Development and Quality Control, Ministry of Agriculture/ National-Local Joint Engineering Laboratory of Se-enriched Food Development (Grant No. Se-2021C08), Major science and technology projects in Huaibei (Grant No. HK2021016), and Industry university research cooperation project of Anhui Kouzi Distillery Co., Ltd. and Huaibei Normal University (Grant No. KZJY-001), National University Student Innovation and Entrepreneurship Project (Grant No. 202210373018).

## Conflict of interest

Authors JX, HL, YZ, LC, SS, and QX were employed by Anhui Kouzi Distillery Co., Ltd. The remaining authors declare that the research was conducted in the absence of any commercial or financial relationships that could be construed as a potential conflict of interest.

## Publisher's note

All claims expressed in this article are solely those of the authors and do not necessarily represent those of their affiliated organizations, or those of the publisher, the editors and the reviewers. Any product that may be evaluated in this article, or claim that may be made by its manufacturer, is not guaranteed or endorsed by the publisher.
