# Supplementary material for: Optimisation of the production of a selenium-enriched polysaccharide from Cordyceps cicadae S1 and its structure and antioxidant activity
Source: Front Nutr. 2022 Oct 20;9:1032289. doi: 10.3389/fnut.2022.1032289 (PMC9631440; doi:10.3389/fnut.2022.1032289)
Supplement: Supplementary file 1 [file Table_1.DOCX]

Table S1 Plackett-Burman experiment and its main effect analysis

| Model term | Coefficient | Coefficient standard error | Effect | T Value | P Value | Importance ranking |
| --- | --- | --- | --- | --- | --- | --- |
| Constant | 4.534 | 0.165 |  | 27.54 | 0.000 |  |
| Sucrose | -0.818 | 0.165 | -1.637 | -4.97 | 0.003 | 2 |
| Yeast powder | -1.008 | 0.165 | -2.016 | -6.12 | 0.001 | 1 |
| Sodium selenite | 0.14 | 0.165 | 0.279 | 0.85 | 0.429 | 4 |
| K^+^ | -0.672 | 0.165 | -1.344 | -4.08 | 0.006 | 3 |
| Mg^2+^ | 0.058 | 0.165 | 0.117 | 0.35 | 0.735 | 5 |

Table S2. Response surface analysis results

| Model term | Coefficient | Coefficient standard error | T Value | P Value |
| --- | --- | --- | --- | --- |
| Constant | 8.354 | 0.274 | 30.47 | 0.000 |
| X1 | -0.019 | 0.168 | -0.11 | 0.915 |
| X2 | 0.017 | 0.168 | 0.10 | 0.921 |
| X3 | 0.047 | 0.168 | 0.28 | 0.790 |
| X1* X1 | -2.387 | 0.247 | -9.66 | 0.000 |
| X2* X2 | -1.931 | 0.247 | -7.82 | 0.001 |
| X3* X3 | -1.654 | 0.247 | -6.69 | 0.001 |
| X1* X2 | -0.168 | 0.237 | -0.71 | 0.510 |
| X1* X3 | -0.174 | 0.237 | -0.74 | 0.495 |
| X2* X3 | 0.303 | 0.237 | 1.28 | 0.258 |

Table S3. Analysis of variance

| Source | Freedom | Adj SS | Adj MS | F Value | P Value |
| --- | --- | --- | --- | --- | --- |
| Model | 9 | 39.7781 | 4.4191 | 19.60 | 0.002 |
| Linear | 3 | 0.0231 | 0.0077 | 0.03 | 0.990 |
| X1 | 1 | 0.0028 | 0.0028 | 0.01 | 0.915 |
| X2 | 1 | 0.0025 | 0.0025 | 0.01 | 0.921 |
| X3 | 1 | 0.0179 | 0.0179 | 0.08 | 0.790 |
| Square | 3 | 39.1461 | 13.0487 | 57.88 | 0.000 |
| X1* X1 | 1 | 21.0438 | 21.9438 | 93.35 | 0.000 |
| X2* X2 | 1 | 13.7725 | 13.7725 | 61.09 | 0.001 |
| X3* X3 | 1 | 10.0991 | 10.0991 | 44.80 | 0.001 |
| Two factor interaction | 3 | 0.6026 | 0.2009 | 0.89 | 0.506 |
| X1* X2 | 1 | 0.1136 | 0.1136 | 0.50 | 0.510 |
| X1* X3 | 1 | 0.1218 | 0.1218 | 0.54 | 0.495 |
| X2* X3 | 1 | 0.3672 | 0.3672 | 1.63 | 0.258 |
| Error | 5 | 1.1272 | 0.2254 |  |  |
| Misfit | 3 | 0.3336 | 0.1112 | 0.28 | 0.839 |
| Pure error | 2 | 0.7935 | 0.3968 |  |  |
| Total | 14 | 40.8990 |  |  |  |
